# Authors' Response to Peer Reviews of “Using Structural Equation Modelling in Routine Clinical Data on Diabetes and Depression: Observational Cohort Study”

**DOI:** 10.2196/38010

**Published:** 2022-04-27

**Authors:** Amy Ronaldson, Mark Freestone, Haoyuan Zhang, William Marsh, Kamaldeep Bhui

**Affiliations:** 1 Wolfson Institute for Preventive Medicine Queen Mary University of London London United Kingdom; 2 School for Electronic Engineering and Computer Science Queen Mary University of London London United Kingdom; 3 Department of Psychiatry Nuffield Department of Primary Care Sciences University of Oxford Oxford United Kingdom

**Keywords:** depression, diabetes, electronic health records, acute care, PLS-SEM, path analysis, equation modelling, accident, emergency care, emergency, structural equation modelling, clinical data

*The author’s response to peer-review reports for “Using Structural Equation Modelling in Routine Clinical Data on Diabetes and Depression: Observational Cohort Study.”*

## Round 1 Review

Thank you for the review of this submission [1] to the Journal of Medical Internet Research. We have considered the comments carefully and have revised the manuscript to address the issues raised. Our responses to the points made by the two reviewers [2,3] are detailed below.

We have submitted a revised version of the manuscript without tracked changes as requested. A copy of the manuscript with tracked changes has been included in the submission as a supplementary file.

### Reviewer CJ [1]

#### General Comments

This paper takes structural equation modelling (SEM) and uses it in a novel way that could be beneficial for researchers and clinicians alike. The results and discussion are transparent, and do not overstate the findings. The researchers created a complex model that could demonstrate the benefits of use of this data analysis method in other health care contexts. The future directions and recommendations are realistic.

#### Specific Comments

##### Major Comments

1. Lacks a statement of the study design. SEM is the method of analysis, not the study design.
  Response: We have now amended the Methods subsection “Data Source and Study Design” to include a statement indicating that this study was a cross-sectional observational cohort study (p4).

##### Minor Comments

1. Write out “A&E” in title and first mention in text of abstract.
  Response: Thank you for pointing this out. We have now amended the title and abstract.
2. In the Introduction and second section, you have 2 statements that are in close proximity and convey similar information. I would consider revising. Introduction statement: “Therefore, we sought to determine whether SEM could be used to make this data set more ‘research friendly’ by attempting to create clinical constructs and model some well-known clinical associations between depression and accident & emergency (A&E) use in patients with type 2 diabetes.” Next section statement: “Therefore, we sought to test whether SEM could be applied to a large routine clinical data set from East London to model these associations between depression, diabetic care, diabetic control, and A&E utilization, while assessing the impact of current mental health care provision.” Perhaps go with the second one.
  Response: Thank you for pointing this out. We agree it is somewhat repetitive and have amended the second statement so that it is now a development of the first statement (p3).
3. Measures of Mental Health Diagnosis and Care - The information on the AUDIT seems misplaced or excessive since other outcome measures are not explained in that amount of detail. Consider removing: “Scores on the AUDIT range from 0-40, with higher scores indicating higher risk of dependence. The AUDIT C consists of the three consumption questions from the AUDIT and scores can range from 0-12, with higher scores indicating higher risk.”
  Response: We agree that we provide what seems to be an excessive description of the alcohol intake measures. This is because the variable itself was complex as the AUDIT and the AUDIT-C were combined in the data set (by the commissioning support unit), which led to two different scales being used to measure the same thing. For full transparency, we feel that we need to include this rather lengthy description in the paper. We believe it also reflects the complexity of using routine clinical data and data linkage.
4. I don't think you need to state this: “A full description of the adult mental health care cluster codes used by the NHS can be found here: (link).” Just state those are the clusters you chose, and why.
  Response: We agree and have now deleted the sentence and link.
5. Data Source: Consider explaining what the intended purpose of each data source/database is. These are largely unknown to anyone outside the UK health care context and will require more detail.
  Response: We agree that more detail is required for non-UK readers and have now provided a more detailed description of the data sources on page 4.
6. More explanation of what partial least squares (PLS) SEM is might be beneficial for the reader.
  Response: We have added some further explanation of PLS-SEM with appropriate introductory references for the nonstatistician on p3 of the manuscript as follows:
  “Structural equation modelling (SEM) is a statistical technique that allows for the inclusion of multiple variables and the creation of important constructs that cannot be observed directly. Partial least squares SEM (PLS-SEM) is a variant of SEM that poses no distributional assumptions (eg, normality, continuous/scale) upon data used for modelling but is frequently used for predictive approaches with an aim to understanding causal structures. Further, PLS-SEM can be effective with a relatively small sample: approximately 10 cases per regression or ‘path’ estimate leading to the most connected latent variable is considered adequate, although there has been some debate about the use of PLS-SEM with very small sample sizes.”
7. May benefit from explanation of why PLS versus covariance-based (CB) and other SEM types since the sample size was large (PLS-SEM is a great choice in my mind, but others may want more justification).
  Response: We have added the following text to help explain our choice of approach on p7.
  “Given the nature of the data, which consisted mainly of dichotomous indicators (eg, diagnoses) and ordinal measures (eg, AUDIT drinking scores) with only a small number of continuous observed variables (eg, HbA1c reading), PLS-SEM was selected over other SEM approaches as it allows for the use of both continuous and discrete observed variables as indicators that measure unobservable latent variables. A covariance-based SEM approach (CB-SEM) would require continuous variables with some restrictions on distribution; Bayesian networks were also considered but are entirely probabilistic in outcome and would not have given the desired effect size coefficients for different pathways.”
8. State whether the structural model is reflexive or formative and justification for this.
  Response: This is a reflective model—we have added the following text on p7:
  “Our modelling approach was reflective, in that we employed observed variables from the health care data set to measure pre-existing latent variables (eg, “A&E usage”) and that, to use the typology proposed by Coltman et al, causality flows from latent construct to observed variable (eg, A&E usage [construct] causes increased spend on A&E services [observed]).”
9. Discussion: there are 2 similar comments in close proximity: “This might be related to a problem with the data set, which will be described later in the Discussion” and “This is not in agreement with previous research, which has shown that improvement of depressive symptoms through the use of psychotherapy and pharmacotherapy is associated with improved glycemic control. The opposite association reported in this study is likely related to issues with data quality, which will be outlined later.”
  Response: We agree this is somewhat repetitive and have removed the first comment from the Discussion as it did not add a huge amount to the interpretation of the data.
10. In the Limitations section, link those statements to the above issue (10) for clarity.
  Response: In the original Limitations section of the Discussion, we do link back to the previous statement when we say the following:
  “The problem with the IAPT data likely affected the mental health treatment latent variable in the SEM and might help to explain why mental health treatment was not associated with poor diabetic control.”
11. A statement in Future Directions and Recommendations could address issues with the data set and what should/could be done to improve this.
  Response: We have now added some extra recommendations about how the data set and data sets like it could be improved:
  “Improvement of data flows (eg, information about use of IAPT services) and more years of data would address issues around lack of temporality and inaccurate findings.”

### Anonymous reviewer

#### Major comments

1. The general research hypothesis should be interpreted and clarified more in the introduction.
  Response: We thank the reviewer for their suggestion and have now provided some clear research hypotheses in the Introduction (p3, p4):
  “We hypothesised that depression would be associated with increased diabetic complications, poor diabetic control, and that both depression and poor diabetic control would be associated with increased utilisation of A&E. We predicted that the receipt of mental health treatment would improve diabetic control.”
2. Please redesign Figure 1 with better quality and interpretations.
  Response: After some thought, we decided to remove Figure 1 from the manuscript as we believe Figure 2 (now Figure 1 in current version) depicts the latent variables and associations between them sufficiently.
3. Recommendations and limitations are absent.
  Response: In the original manuscript, we provided an extensive account of study limitations in the Discussion section (p13). We also provided a number of recommendations (p14).

#### Minor comments

1. Order keywords alphabetically.
  Response: We have now amended this.

## Abbreviations

CB-SEM: covariance-based structural equation modelling
PLS-SEM: partial least squares structural equation modelling
SEM: structural equation modelling
